# A review on comparative analysis of marine and freshwater fish gut microbiomes: insights into environmental impact on gut microbiota

**DOI:** 10.1093/femsec/fiae169

**Published:** 2024-12-24

**Authors:** Binoy Kumar Singh, Kushal Thakur, Hishani Kumari, Danish Mahajan, Dixit Sharma, Amit Kumar Sharma, Sunil Kumar, Birbal Singh, Pranay Punj Pankaj, Rakesh Kumar

**Affiliations:** Department of Animal Sciences, School of Life Sciences, Central University of Himachal Pradesh, Dharamshala 176206, India; Department of Animal Sciences, School of Life Sciences, Central University of Himachal Pradesh, Dharamshala 176206, India; Department of Animal Sciences, School of Life Sciences, Central University of Himachal Pradesh, Dharamshala 176206, India; Department of Animal Sciences, School of Life Sciences, Central University of Himachal Pradesh, Dharamshala 176206, India; Department of Animal Sciences, School of Life Sciences, Central University of Himachal Pradesh, Dharamshala 176206, India; Department of Animal Sciences, School of Life Sciences, Central University of Himachal Pradesh, Dharamshala 176206, India; Department of Animal Sciences, School of Life Sciences, Central University of Himachal Pradesh, Dharamshala 176206, India; ICAR—Indian Veterinary Research Institute (IVRI), Regional Station, Palampur 176061, India; Department of Zoology, Nagaland University (A Central University), Lumami 798627, India; Department of Animal Sciences, School of Life Sciences, Central University of Himachal Pradesh, Dharamshala 176206, India

**Keywords:** gut microbiome, freshwater fishes, marine fishes, environmental factors, comparative analysis: aquaculture

## Abstract

The gut microbiota, which includes prokaryotes, archaea, and eukaryotes such as yeasts, some protozoa, and fungi, significantly impacts fish by affecting digestion, metabolism, and the immune system. In this research, we combine various tasks carried out by various bacteria in the gut of fish. This study also examines the gut microbiome composition of marine and freshwater fish, identifying important bacterial species linked to different biological functions. The diversity within fish species highlights the importance of considering nutrition, habitat, and environmental factors in microbiological research on fish. The ever-changing gut microbiome of the fish indicates that microbial communities are specifically adapted to meet the needs of both the host and its environment. This indicates that the fish can adjust to a specific environment with the help of gut microbiota. This important research is crucial for comprehending the complex relationships between fish and their gut bacteria in different aquatic environments. These discoveries have implications for aquaculture practices, fisheries administration, and the broader ecological processes of both freshwater and marine environments. With further progress in this area of study, the knowledge acquired would offer a valuable standpoint to enhance our comprehension of aquatic microbiology and enhance the sustainability and nutrition of fish resources.

## Teaser

The comparative analysis of marine and freshwater fish gut microbiomes reveals significant differences in microbial composition and diversity, highlighting the influence of distinct environmental factors on gut microbiota.

## Introduction

The gut of an animal consists of trillions of diverse microorganisms that can have both positive and negative effects on the nutrition, immunity, and overall well-being of the host (Bairagi et al. 2002, Ray et al. 2012, Deb et al. 2020, Ghori et al. 2022, De Marco et al. 2023). Its structure is influenced by factors such as microbial diversity, spatial distribution, pH, and interactions with host enzymes (Jordaan and Bezuidenhout 2013, Kim et al. 2021). Microbes in the gut of fish engage in competition, generate antimicrobials, communicate, and consume each other, impacting the population dynamics and health of the host (Wang et al. 2018, Cui et al. 2022, Luan et al. 2023). Struggles between microorganisms, such as competition for resources and bacteriophage assaults, impact the equilibrium of microbes (Di Maiuta et al. 2013, Parris et al. 2019, Qi et al. 2023b). The fish has a unique assemblage of microorganisms residing within their gastrointestinal tract (Givens et al. 2015, Deb et al. 2020, Zou et al. 2020, Xi et al. 2023). Some of these microorganisms form a dynamic and symbiont relationship with the host and impact various aspects of fish biology such as digestion, absorption, synthesis of essential nutrients, antimicrobial peptides (AMPs), and bacteriocins’ cellular and humoral immunity (Roeselers et al. 2011, De Marco et al. 2023). In return, the host receives exogenous enzymes and nutrients, such as vitamins and fatty acids, which cannot be produced by the host body cells (Dhanasiri et al. 2011, Wu et al. 2024). A balanced microbiome composition reduces the colonization and proliferation of harmful pathogens and controls diseases (Fjellheim et al. 2007, Ou et al. 2024). Therefore, the microbiota of the gut is considered an “extra organ” owing to powerful microbial genes, and the role of microorganisms in digestion, immunity, and overall development (Bairagi et al. 2002, Dhanasiri et al. 2011, Feng et al. 2018, Butt and Volkoff 2019). Facultative anaerobes and aerobes are present in greater numbers in the fish gut in comparison to obligate anaerobes (Cahill 1990, Clements 1997, Izvekova et al. 2007, Trust et al. 2011). This is mainly because of the fish gut environment, which typically has higher oxygen levels, particularly in the front parts such as the stomach and nearby intestine (Nelson and Dehn 2010, Egerton et al. 2018). Facultative anaerobes can adjust to changing levels of oxygen, while obligate anaerobes prefer environments with no oxygen, such as the lower regions of mammalian intestines (André et al. 2021, Lu and Imlay 2021, Duncan et al. 2023). The gut microbiomes are divided into autochthonous (i.e. native bacteria or when they can attach and colonize the gut epithelial surface of the host) and allochthonous (i.e. foreign bacteria or when they accidentally enter the host gut and get removed after some time without colonizing) (Nayak 2010, Navarrete et al. 2013, Givens et al. 2015, Sharma et al. 2023). Therefore, a thorough understanding of the fish gut microbiome is very important in aquaculture because it can be helpful in the management of fisheries and conservation and has the potential to boost fish health and sustainable seafood production (Van Kessel et al. 2011). In aquaculture, it is crucial to preserve a balanced gut microbiome to prevent diseases that could have a severe impact on fish populations. Probiotic and prebiotic therapies are frequently employed to improve advantageous microbial populations, resulting in improved feed efficiency and decreased expenses in fish farming (Merrifield et al. 2010, Dutta 2015, Ghori et al. 2022, De Marco et al. 2023). Moreover, a well-balanced gut microbiome can aid in decreasing waste generation, thereby lessening the environmental consequences of aquaculture operations. According to Miranda et al. (2022), numerous fish species are at risk of extinction because of human activities and climate change, and yet little is known about their microbiota, making the study of intestinal microbiota crucial for the conservation of these species (Soh et al. 2024). The gut microbiome is crucial for the health and survival of fish, particularly in captive breeding programs aimed at species conservation (West et al. 2019, Ruiz et al. 2024). A properly cared for microbiome helps fish adjust to shifting environmental conditions, especially crucial with climate change and habitat damage. Having a strong gut microbiome can boost the chances of survival for fish being released back into their natural habitat by enhancing their overall health and ability to fight off diseases. Comparative analysis of the fish gut microbiome is an important field of research as it can unravel hidden realities that can help to understand the relations between microorganisms, and microbial interaction with their host besides functions and diversity of the complex microbiota.

The function and composition of the microbiome may vary from species to species like other aquatic and terrestrial animals (Sehnal et al. 2021, de Jonge et al. 2022). This intriguing scientific endeavour involves studying and comparing the composition, diversity, and functional roles of these microbial communities across a wide spectrum of fish, ranging from freshwater to marine species, and from herbivorous to carnivorous feeders (Givens et al. 2015). Thus, we aimed to gain a deeper understanding of how microbial communities have evolved in response to the specific dietary, environmental, and physiological adaptations of fish species. Through comparative analysis, researchers have uncovered the profound impact of the fish gut microbiome on various aspects of fish biology, including immunity, metabolism, growth, and even behaviour (Collazos et al. 1994, Aquac et al. 2023). The insights obtained from this study will not only contribute to our understanding of fish health and ecology, but also have immense promise for enhancing aquaculture practices, conserving endangered species, and advancing gut microbial biotechnological applications (Ghanbari et al. 2015).

**Figure 1.**
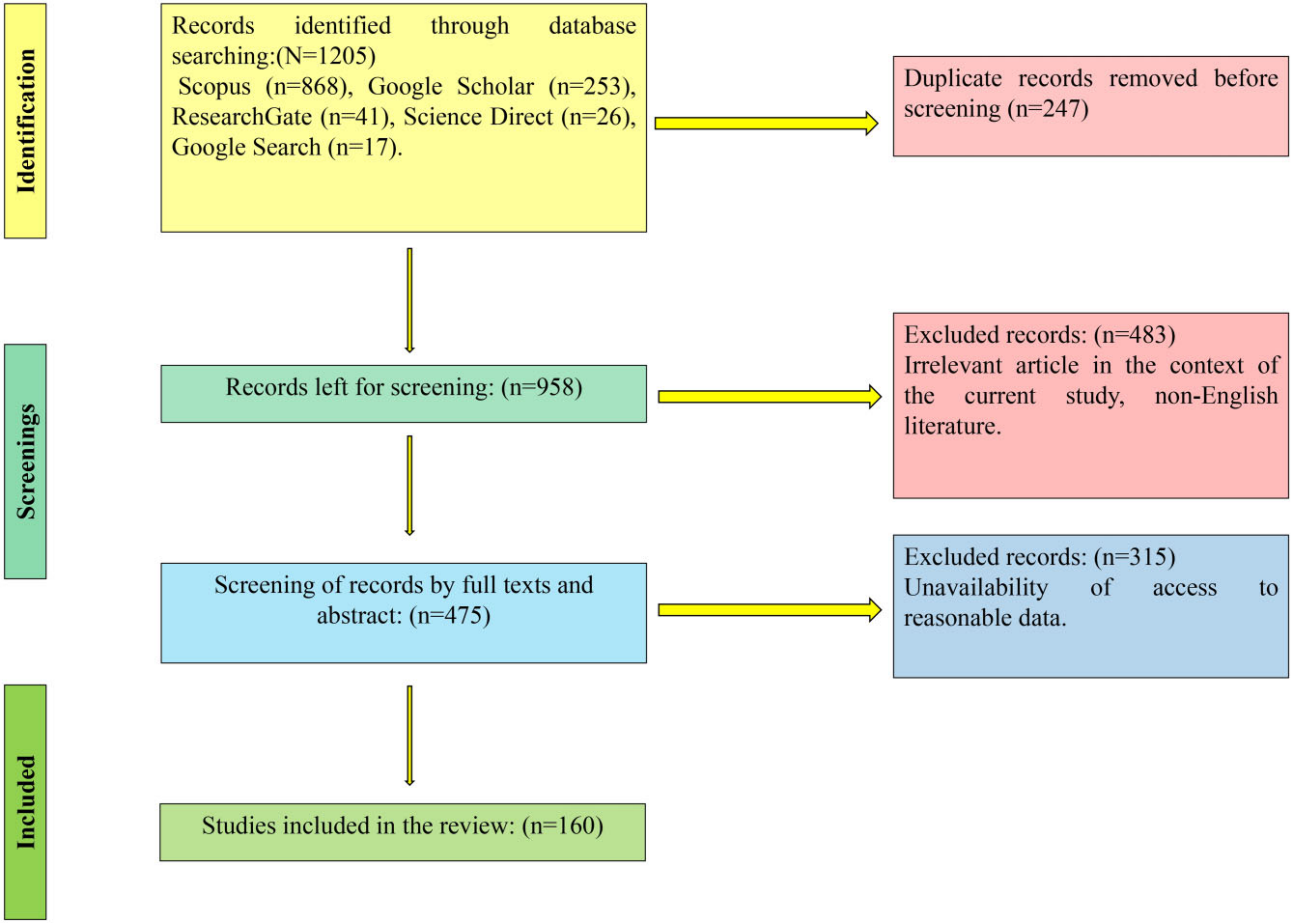
PRISMA methodology was followed during the literature survey.

Thorough research was conducted using appropriate keywords on online platforms such as Google Scholar, ResearchGate, Science Direct, Scopus, and regular Google searches to find accurate data. The Preffered Reporting Items for Systematic Reviews and Meta-Analysis (PRISMA) methodology used for systematic review has been depicted in Fig. 1. Certain pertinent articles that were connected to the keywords and subject have been incorporated in the research. Articles that are not pertinent, lack crucial information, are not in full text, and are off-topic were eliminated. Most of the literature examined was from the years 2015 to 2024, with some older literature included due to incomplete data.

### Functional status of fish gut microbiome

**Figure 2.**
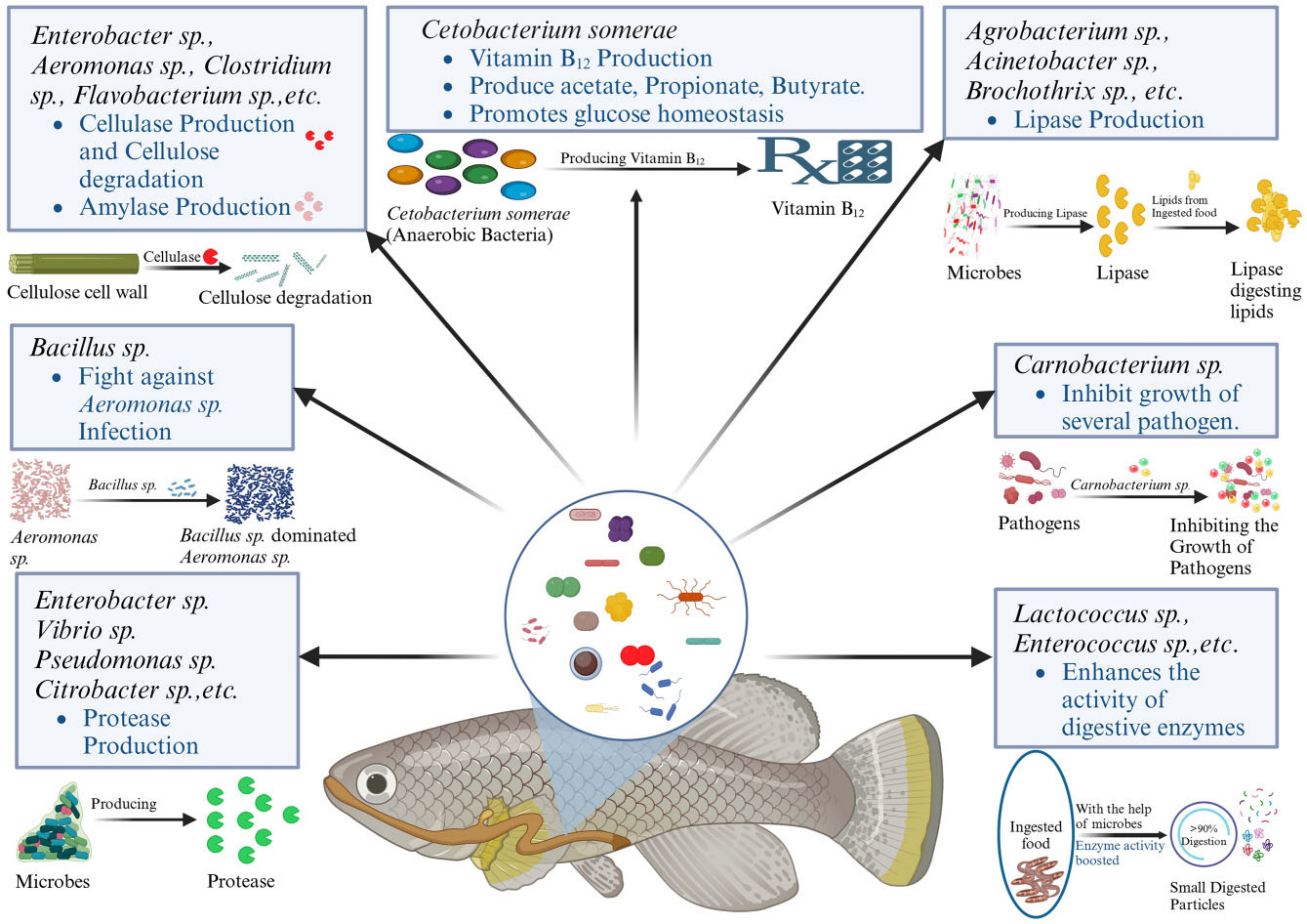
Function of microorganisms in fish gut. Some bacteria showing probiotic and pro-health benefits have been highlighted. Microbial feed additives (probiotics and synbiotics) are available commercially to improve fish nutrition and health.

The study on fish is increasing progressively due to the demand for fish and fish-origin nutraceuticals The gut microbiota plays a crucial role in regulating the growth and production of fish, hence aiding in meeting the market demand for fish (Sullam et al. 2012, Wong et al. 2013, Butt and Volkoff 2019, Liu et al. 2021). Proper knowledge of bacterial function in a particular species of fish can help us develop efficient probiotic strains or synbiotics (as depicted in Fig. 2). For instance, *Cetobacterium somerae*, a Gram-negative micro aerotolerant bacterium present in the gastrointestinal tract (GI) tract of some freshwater fish such as tilapia and carp, produces large amounts of Vitamin B_12_ (Tsuchiya et al. 2008)_._ The fish harbouring *C. somerae* in their gut, in general, did not require Vitamin B_12_ in their diet, whereas species such as catfish and Japanese eel that do not have *C. somerae* in their gut require Vitamin B_12_ (Tsuchiya et al. 2008, Jobling 2012). *Cetobacterium somerae* is crucial for fish, particularly those consuming plant-based diets or having low B_12_ levels, as it aids in protein fermentation and amino acid absorption for their growth and energy needs (Sugita et al. 1991, Li et al. 2015). It generates a large quantity of acetate, contributing to improved glucose regulation, enhanced gut barrier function, and increased resistance to diseases (Wang et al. 2021, Qi et al. 2023b). As a prevalent gut bacterium, it helps support a balanced ecosystem by beating harmful bacteria and creating substances such as short-chain fatty acids (SCFAs) (Sugita et al. 1991, Li et al. 2015, Bhardwaj et al. 2023). While it has positive effects on fish, the impact on human health in aquaculture environments is not clearly understood, and there may be risks of transmission (Finegold et al. 2003). The generation of gases such as hydrogen and methane in the process of fermentation is also a feature of *C. somerae* (Li et al. 2015). In general, this bacterium plays a role in supporting gut health, energy metabolism, and overall well-being in fish such as carp, tilapia, and catfish, underscoring its significance in keeping a balanced and healthy gut microbiome. More studies are required to comprehend how it could affect human health, and the dangers linked to its existence in aquaculture.

Many microbes are involved in the digestion process (Ray et al. 2012, Karasov and Douglas 2013, Ringø et al. 2016, Sehnal et al. 2021). The function is more clear in herbivorous and omnivorous fish that eat diets with cellulose and plant secondary compounds such as tannins, alkaloids, and flavonoids (Nelson et al. 1999, Francis et al. 2001, Li et al. 2016). Specific microbial communities are needed to break down complex carbohydrates and detoxify secondary metabolites. Bacteria such as *Aeromonas sobria, A. veronii, A. hydrophila, A. jandaei, Enterobacter aerogenes, E. ludwigii, Clostridium* sp., *Citrobacter braakii, Raoultella ornithinolytica, Klebsiella variicola, Pseudomonas veronii, Erwinia billingiae, Enterococcus faecium, Brevibacillus laterosporus, Anoxybacillus* sp., *Bacillus megaterium*, and *Sediminibacterium salmoneum* provide the necessary enzymes for plant-based diets (Bairagi et al. 2002, Saha et al. 2006, Ray et al. 2012, Ye et al. 2014, Li et al. 2016). The microbial consortia vary according to the host species, diet, habitat and environmental factors (Kumar et al. 2023). Some of the bacteria producing fibrolytic enzymes, reported from different fishes, have been summarized in Table 1. Many carnivorous fish feed on crustaceans that are digested by chitinase-producing gut bacteria such as *Marinobacter lutaoensis, Pseudoalteromonas piscicida, Pseudomonas* spp., *Ferrimonas balearica, Enterovibrio norvegicus, Grimontia hollisae, Photobacterium damselae* spp., *Acinetobacter* spp., *Vibrio* spp., *Enterobacter* spp., *Aeromonas* spp., *Flavobacterium* spp., and *Photobacterium* spp. (Ray et al. 2012).

**Table 1.** List of microbes along with its functions reported by different researchers who conducted studies in different fishes.

Sl. No.	Microbes	Functions/role	References
1	*Aeromonas sobria, A. veronii, A. hydrophila, A. jandaei, Enterobacter* sp., *E. aerogenes, E. ludwigii, Clostridium* sp., *Citrobacter braakii, Raoultella ornithinolytica, Klebsiella variicola, Pseudomonas veronii, Erwinia billingiae, Enterococcus faecium, Brevibacillus laterosporus, Anoxybacillus* sp., *Bacillus megaterium, Sediminibacterium salmoneum*	Cellulose degradation	(Bairagi et al. 2002, Saha et al. 2006, Ray et al. 2012, Wu et al. 2012, Ye et al. 2014, Li et al. 2016)
2	*Cetobacterium somerae*	Synthesizes Vitamin B_12_; produces acetate, propionate, and butyrate; and promotes glucose homeostasis	(Kim et al. 2021, Wang et al. 2021, Qi et al. 2023a)
3	*Lactococcus lactis* and *Enterococcus faecalis*	Enhance the activity of digestive enzyme	(Luan et al. 2023)
4	*Lactococcus lactis*	Promotes an increase in beneficial microbes and decrease pathogenic bacteria	(Luan et al. 2023)
5	*Bacillus cereus* and *B. thuringiensis*	Function against *Aeromonas hydrophila* infection	(Kong et al. 2021, Luan et al. 2023)
6	*Carnobacterium* sp.	Inhibits several pathogens	(Nayak 2010)
7	*Aeromonas hydrophila, Aeromonas* spp., *Bacteroidaceae, Clostridium* spp., *Bacillus circulans, B. pumilus, B. cereus, Aeromonas* spp., *Enterobacteriaceae, Pseudomonas* spp., *Flavobacterium* spp., *Citrobacter freundii, B. subtilis, Brochothrix* sp., *Brochothrix thermosphacta*	Amylase production	(Bairagi et al. 2002, Ray et al. 2012)
8	*Enterobacter* spp., *Vibrio* spp., *Pseudomonas* spp., *Acinetobacter* spp., *Aeromonas* spp., *Flavobacterium balustinum, Bacillus cereus, B. circulans, B. pumilus, Citrobacter* sp., *Citrobacter freundii, B. licheniformis, B. subtilis*	Protease production	(Bairagi et al. 2002, Ray, Ghosh and Ringø 2012)
9	*Agrobacterium* sp., *Brevibacterium* sp., *Microbacterium* sp., *Staphylococcu* sp., *Vibrio* spp., *Acinetobacter* spp., *Enterobacteriaceae, Pseodomonas* spp., *Bacillus thuringiensis, B. cereus, Bacillus* sp., *Brochothrix* sp., *Brochothrix thermosphacta*	Lipase production	(Bairagi et al. 2002, Ringø et al. 2010, Ray et al. 2012)
10	*Marinobacter lutaoensis, Ferrimonas balearica, Pseudoalteromonas piscicida, Enterovibrio norvegicus, Grimontia hollisae, Photobacterium damselae* spp. *damselae, P. leiognathi, P. lipolyticum, P. phosphoreum, P. rosenbergii, Vibrio campbelli, V. chagasii, V. fischeri, V. fortis, V. gallicus, V. harveyi, V. natrigenes, V. nigripulchritudo, V. ordalii, V. parahaemolyticus, V. pomeroyi, V. ponticus, V. proteolyticus, V. rumoiensis, V. shilonii, V. tasmaniensis* and *V. tubiashii, Enterobacter* spp., *Vibrio* spp., *Pseudomonas* spp., *Aeromonas* spp., *Vibrio* spp., *Acinetobacter* sp., *Enterobacteriaceae, Flavobacterium* sp., *Photobacterium* spp.	Chitinase production	(Ray, Ghosh and Ringø 2012)
11	*Streptococcus* sp., *Leuconostoc* sp., *Pediococcus* sp., *Aerococcus* sp., *Enterococcus* sp., *Vagococcus* sp., *Carnobacterium* sp., *Carnobacterium divergens, C. piscicola, Lactobacillus* spp., *L. plantarum, L. rhamnosus, L. bulgaricus*	Lactic acid fermentation and produce organic acids, hydrogen peroxide, and some other substances suppressing the growth of pathogenic microorganisms	(Ringø and Gatesoupe 1998, Gatesoupe 2007, Izvekova et al. 2007, Ringø et al. 2010)

Various studies also reported about microbes fighting against harmful bacteria and diseases such as *Streptococcus* sp., *Pediococcus* spp., *Aerococcus* spp., *Enterococcus* spp., *Vagococcus* spp., *Carnobacterium* spp., *Lactobacillus* spp., and *Bacillus* spp., *Leuconostoc* spp., and *Lactococcus lactis* (Ringø and Gatesoupe 1998, Gatesoupe 2007, Izvekova et al. 2007, Nayak 2010, Ringø et al. 2010, Kong et al. 2021, Luan et al. 2023). Various types of bacteria found in the intestines create diverse bioactive substances and specific genes that play a crucial role in the production of secondary metabolites. In Table 2, some of the important genes are presented that are known to play a role in producing secondary metabolites within the microbiome of fish guts. Genes, including *cobA, cobG*, and *cobT*, are essential for synthesizing Vitamin B_12_ in fish and can be found in *Cetobacterium somerae, Clostridium* spp., and *Propionibacterium* spp. (Fang et al. 2017, Guo and Chen 2018, Balabanova et al. 2021, Qi et al. 2023b). The production of SCFAs, such as butyrate and propionate, depends on genes such as *but* and *buk* present in *Clostridium* and *Bacteroides* species (Vital et al. 2014, Tarnecki et al. 2017, Meng and Shu 2024). Fish use genes such as *iucA* and *pvd* from *Pseudomonas* and *Vibrio* species for siderophore biosynthesis to acquire iron (Ravel and Cornelis 2003, Hassan and Troxell 2013, Mydy et al. 2020). The production of antimicrobial peptides such as lantibiotics and nisin is crucial for preserving a balanced microbial population in fish, and it involves genes such as *lantA* and *nisA* that are present in *Lactobacillus* and *Bacillus* species (Siegers and Entian 1995, McAuliffe et al. 2001, Kuipers et al. 2011, Egerton et al. 2018). *PKS* and *NRPS* pathways in marine *Streptomyces* and *Pseudomonas* species synthesize secondary metabolites such as antibiotics and pigments, with regulation by *PKS* gene clusters. *NRPS* gene clusters are in charge of creating non-ribosomal peptides, which have the ability to serve as antibiotics or signalling molecules, impacting both microbial competition and fish health (Ray et al. 2012, Wang et al. 2014, Borsetto et al. 2019, Komaki et al. 2020, Yin et al. 2023b). Molecules involved in quorum sensing, such as acyl-homoserine lactones (AHLs) produced by genes *luxI* and *luxR*, enable bacteria such as*Vibrio, Aeromonas*, and *Pseudomonas* to regulate activities such as biofilm formation and virulence factors (Miyashiro and Ruby 2012, Rajput and Kumar 2017). The *tnaA* gene encodes tryptophanase, which aids bacteria such as *Escherichia coli* and *Lactobacillus* in generating indole and its derivatives that impact gut barrier integrity and inflammation (Li and Young 2013, Boya et al. 2021). The genes *cysJIH* found in organisms such as *Desulfovibrio* play a role in generating hydrogen sulphide, which can exhibit anti-inflammatory properties when present in small amounts (Ostrowski et al. 1989, Álvarez et al. 2015). The *srfA* operon found in *Bacillus* and *Pseudomonas* helps in the production of biosurfactants, which support bacterial colonization and prevent biofilm formation (Kisil et al. 2023, Xu et al. 2023, Qi et al. 2023a). Genes involved in terpenoid biosynthesis, such as *dxs* and *ispG*, are responsible for signalling and possible antimicrobial roles in the GI tract of *Streptomyces* and *Cyanobacteria* (Xue et al. 2015, Marshall et al. 2023). These procedures demonstrate the various crucial functions that bacterial genes and molecules have in maintaining gut health and communication. In general, these processes are crucial for the well-being of fish, their energy metabolism, the health of their digestive system, and their defence against infections, demonstrating the complex relationships between microbes and genetic processes in aquatic settings.

**Table 2.** Some of the important genes known to play a role in the creation of secondary metabolites in the fish gut microbiome.

Metabolites	Key genes	Bacteria	Function	Reference
**Vitamin B_12_ (cobalamin)**	*cobA, cobG, cobL, cobM, cobT*	*Cetobacterium, Clostridium*	Cobalamin biosynthesis (DNA synthesis, metabolism)	(Fang et al. 2017, Guo and Chen 2018, Balabanova et al. 2021, Qi et al. 2023b)
**Short-chain fatty acids**	*but, buk*, propionate CoA-transferase	*Clostridium, Bacteroides*	Butyrate, propionate, and acetate production (gut health)	(Vital et al. 2014, Tarnecki et al. 2017, Meng and Shu 2024)
**Siderophores**	*iucA, iucB, pvd*	*Pseudomonas, Aeromonas*	Iron acquisition via siderophore production	(Ravel and Cornelis 2003, Hassan and Troxell 2013, Mydy et al. 2020)
**Antimicrobial peptides**	*lantA, lantB, lantC, nisA*	*Lactobacillus, Bacillus*	Bacteriocin production (inhibition of pathogens)	(Siegers and Entian 1995 1995, McAuliffe et al. 2001, Kuipers et al. 2011, Egerton et al. 2018)
**Polyketides/NRPs**	*PKS, NRPS*	*Streptomyces, Bacillus*	Production of antibiotics and immunomodulatory compounds	(Ray et al. 2012, Wang et al. 2014, Borsetto et al. 2019, Komaki et al. 2020, Yin et al. 2023a)
**Quorum sensing**	*luxI, luxR*	*Vibrio, Pseudomonas*	Bacterial communication (biofilm formation, colonization)	(Miyashiro and Ruby 2012, Rajput and Kumar 2017)
**Indole (tryptophan)**	*tnaA*	*E. coli, Lactobacillus*	Gut health regulation and anti-inflammatory signalling	(Li and Young 2013, Boya et al. 2021)
**Hydrogen sulphide**	*cysJIH*	*Desulfovibrio, Clostridium*	Sulphate reduction (gut signalling, motility)	(Ostrowski et al. 1989, Álvarez et al. 2015)
**Biosurfactants**	*srfAA, srfAB, srfAC*	*Bacillus, Pseudomonas*	Surfactin production (colonization, biofilm inhibition)	(Kisil et al. 2023, Qi et al. 2023a, Xu et al. 2023)
**Terpenoids**	*dxs, ispD, ispG*	*Streptomyces, Cyanobacteria*	Signalling molecules and antimicrobial functions	(Xue et al. 2015, Marshall et al. 2023)

The fish intestinal microbiome is an intricate network of symbiotic connections between the host and its microbial residents, encompassing mutualistic, commensal, and antagonistic relationships (Ray et al. 2012). Bacteria play a crucial role by breaking down complex nutrients and producing essential nutrients for the fish, showing the importance of nutrient availability and metabolism (Nayak 2010). The immune system of the host helps tolerate helpful bacteria and inhibits the growth of harmful bacteria (Bledsoe et al. 2022). Quorum sensing enables bacterial populations to communicate and synchronize behaviours such as forming biofilms (Miyashiro and Ruby 2012, Rajput and Kumar 2017, Moreno et al. 2024). Microorganisms compete and spread out in the gut, leading to niche separation, where various bacteria inhabit specific regions and carry out unique functions (Melo-Bolívar et al. 2019). Biofilm development on the intestinal lining provides protection for the host and bacteria against stressors and pathogens (Harika et al. 2020). In general, the microbiome of fish intestines is a constantly changing setting where different types of microbes engage in a fragile equilibrium of collaboration and rivalry.

Antagonistic relationships within the gut microbiome are crucial for upholding microbial equilibrium and hindering the excessive growth of harmful bacteria. These interactions involve the creation of antimicrobial substances such as bacteriocins and organic acids, which hinder the growth of other bacteria (Egerton et al. 2018). Competition for nutrients and space is another factor, as helpful bacteria outcompete harmful ones for limited resources and sites on the gut lining (Tarnecki et al. 2017). Furthermore, bacterial siderophore competition entails the production of molecules to procure iron, which restricts the proliferation of rival organisms (Nayak 2010). Quorum quenching is a different process in which specific bacteria break down signalling molecules produced by pathogens, interrupting their communication and decreasing their ability to cause harm (Rajput and Kumar 2017). In general, these hostile interactions contribute to supporting gut health by preserving a varied and well-balanced microbiome.

### Comparative study of fish gut microbiome: freshwater versus marine water

Marine and freshwater fish have distinct gut microbiomes, influenced by the different environments (Li et al. 2017). Studies reveal that the gut microbiomes of freshwater fish and marine fish are dominated by the phyla Fusobacteria and Proteobacteria (Givens et al. 2015, Li et al. 2017, Deb et al. 2020). Common microbial species found in freshwater fish include Proteobacteria such as *Aeromonas, Pseudomonas*, and *Enterobacter*, Firmicutes such as *Lactobacillus* and *Streptococcus*, Actinobacteria, including *Micrococcus*, and Bacteroidetes such as *Flavobacterium* and *Chryseobacterium* (Sullam et al. 2012, Wu et al. 2012, Llewellyn et al. 2014, Givens et al. 2015, Deb et al. 2020). Marine fish often contain Proteobacteria species such as *Vibrio, Photobacterium*, and *Shewanella*, as well as Firmicutes, including *Bacillus* and *Clostridium*, and Bacteroidetes such as *Cytophaga* (Llewellyn et al. 2014, Givens et al. 2015, Egerton et al. 2018, Ou et al. 2021, Uniacke-Lowe et al. 2024). Planctomyces species, specifically *Planctomycetes*, are marine microorganisms with unique metabolic abilities such as anaerobic ammonium oxidation (Fuerst and Sagulenko 2011). The improvements in high-throughput sequencing techniques have led to the discovery of previously uncultured or poorly understood species in the digestive systems of freshwater and marine fish (Ghanbari et al. 2015, Rasmussen et al. 2022, Brar et al. 2023). A few instances include *Cetobacterium somerae*, which synthesizes Vitamin B_12_ in freshwater fish (Sugita et al. 1991); *ZOR0006*, discovered in carp and tilapia aiding in nutrient uptake (Zhou et al. 2023); and *Endozoicomonas* spp. in marine fish promoting gut health and immunity (Neave et al. 2016). *Aliivibrio* and *Pseudoalteromonas* species have important functions in the gut of marine fish, being involved in bioluminescence and interactions with the host, respectively (Klemetsen et al. 2021, Drønen et al. 2022). Researchers are still studying Tenericutes found in the intestines of marine fish to understand their ecological role as bacteria with smaller genomes, potentially adapted to live in hosts (Givens et al. 2015, Egerton et al. 2018). These results highlight the diverse and important bacteria present in fish guts across different environments.

**Figure 3.**
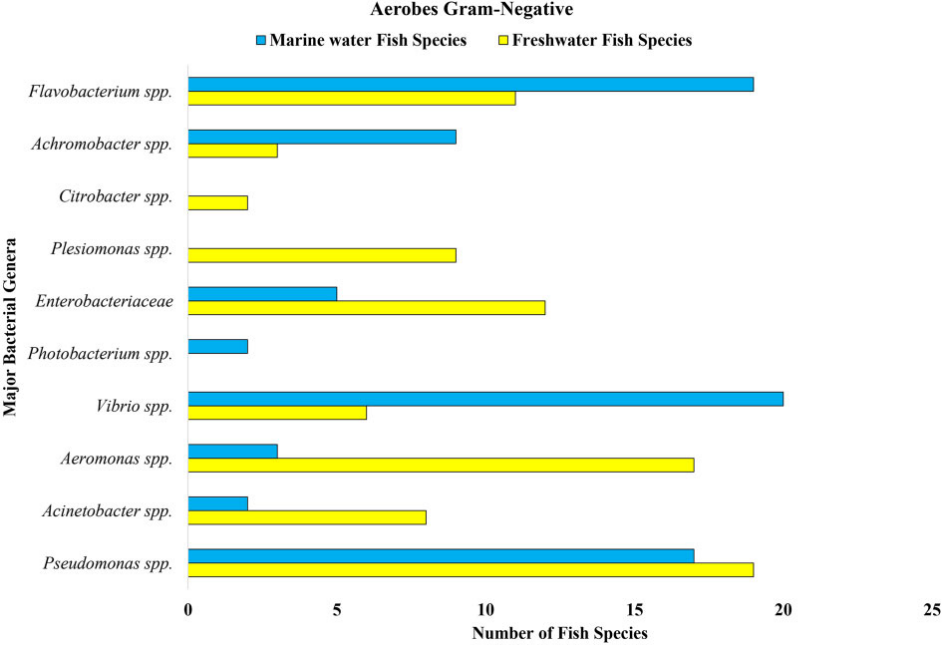
Aerobic Gram-negative bacteria reported in the gut of wild freshwater and marine water fish.

**Figure 4.**
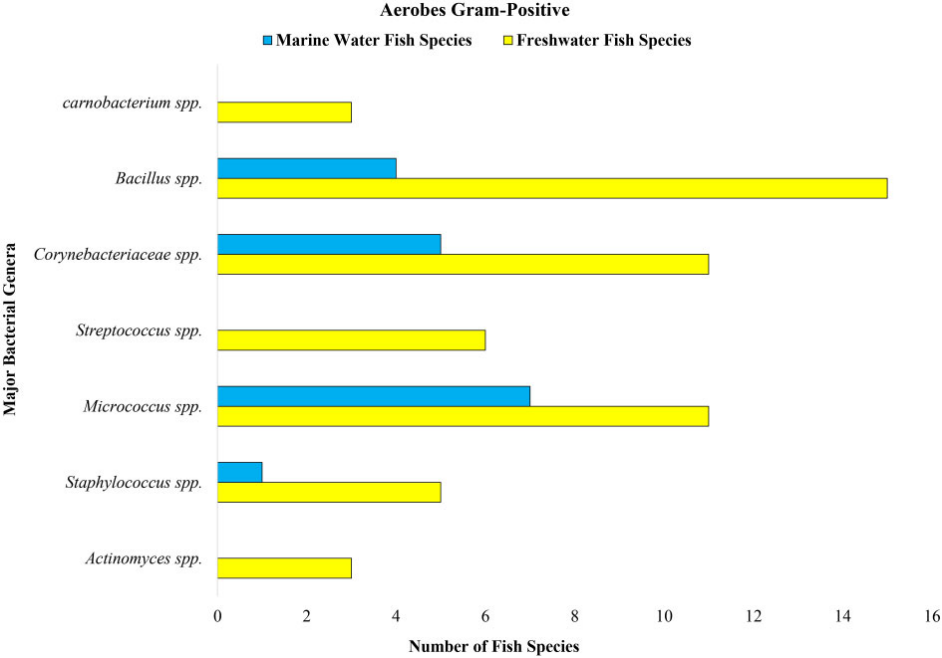
Aerobic Gram-positive bacteria reported in the gut of wild freshwater and marine water fish.

**Figure 5.**
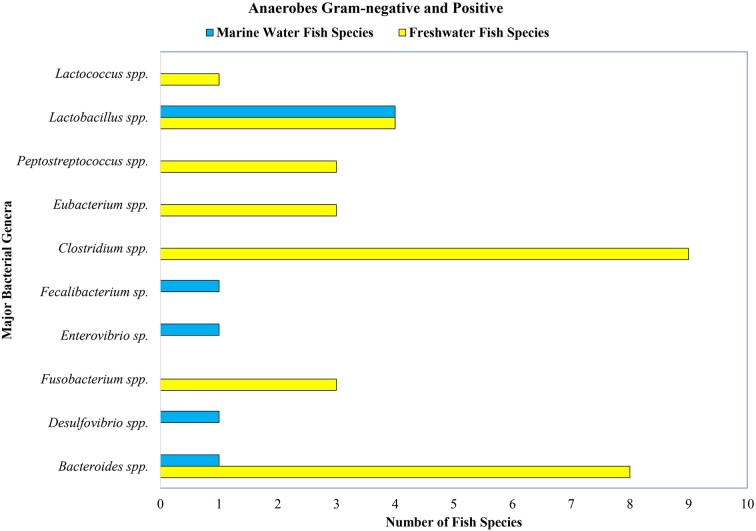
Anaerobic bacteria reported in the gut of wild freshwater and marine water fish.

Some of the reported bacterial groups in marine and freshwater fish guts are presented in Figs 3, 4, and 5. According to Izvekova et al. (2007), these data are obtained as a result of isolation and identification by traditional techniques. The figures include only the popular groups whose composition varies according to habitat. In Fig. 3, it is found that the dominant aerobic Gram-negative bacteria of marine fish are *Flavobacterium* spp., *Achromobacter* spp., *Photobacterium* spp., *Vibrio* spp., and *Pseudomonas* spp., which proves the variation in the gut of marine and freshwater fish. Similarly, the aerobic Gram-positive bacterial data presented in Fig. 4 show that *Bacillus* spp., *Cornebacteriaceae* spp., *Streptococcus* spp., *Lactococcus* spp., *Micrococcus* spp., *Staphylococcus* spp., *Actinomyces* spp., and *Carnobacterium* spp. are present more in freshwater fish than in marine fish (Izvekova et al. 2007). Surprisingly, more anaerobes have been reported in freshwater fish, i.e. *Eubacterium* spp., *Peptostreptococcus* spp., *Fusobacterium* spp., *Clostridium* spp., and *Bacteroides* spp., than in marine fish (depicted in Fig. 5). This might be due to insufficient studies conducted on marine fish and the difficulty involved in isolating anaerobic bacteria. The reason for sharing these data is to demonstrate the difference in gut microbiome among fish living in various environments. Researchers compared the gut microbiomes of 51 fish species and found that 47 species had Gram-negative aerobes, 34 species had Gram-positive aerobes, 10 species had Gram-negative anaerobes, and 8 species had Gram-positive anaerobes (Table S1) (Izvekova et al. 2007). Nevertheless, with the recent development of techniques such as next-generation sequencing, pyrosequencing, etc. (Van Kessel et al. 2011, Terova et al. 2018), we now have a more reliable option for obtaining authentic data. Traditional methods such as isolation and identification, although time-consuming and laborious, still provide a basic understanding of microorganism composition and diversity. Gram-negative bacteria were found in more species and at similar rates in both freshwater and marine fish. Greater quantities of Gram-positive aerobic bacteria were present in freshwater fish and were also observed to host anaerobic bacteria, as depicted in Figs 3, 4, and 5. In order to gain more insight, we also contrasted certain data from freshwater and marine fish presented in Table 3.

**Table 3.** Presence of top bacteria in freshwater and marine water fish species.

Fish species		Environment	Method of study	Diet	Dominant microbiota	Most abundant bacteria	Key functions	References
**Freshwater**	*Onchorhyncus mykiss* (rainbow trout)	Farm	Whole shotgun metagenomic	Omnivorous (insect, plants)	Proteobacteria, Firmicutes, Actinobacteria	*Mycoplasma, Cetobacterium, Lactococcus, Lactobacillus, Leuconostoc, Ureaplasma, Propionibacterium*	Protein digestion, fermentation of carbohydrates	(Nayak 2010, Llewellyn et al. 2014a, Tarnecki et al. 2017, Betiku et al. 2023)
	*Ctenopharyngodon idellus* (grass carp)	Farm	Pyrosequencing	Omnivorous (plants, detritus)	Firmicutes, Bacteroidetes, Cetobacterium	*Streptococcus, Lactobacillus, Flavobacterium, Veillonella, Streptococcus, Pseudomonas, Anoxybacillus, Citrobacter, Clostridium, Leuconostoc*	Fermentation of polysaccharides, Vitamin B_12_ synthesis	(Nayak 2010, Wu et al. 2012, Llewellyn et al. 2014b, Li et al. 2015, Tarnecki et al. 2017)
**Marine Water**	*Salmo salar* (Atlantic salmon)	Farm	High-throughput sequencing	Carnivorous (fish, invertebrates)	Proteobacteria (*Vibrio*), Firmicutes	*Janthinobacterium, Propionibacterium, Stenotrophomonas, Pseudomonas, Phyllobacterium, Delftia, Herbaspirillum, Burkholderia, Sphingomonas, Ochrobactrium, Variovorax, Microbacterium, Rhodococcus, Acinetobacter*	Protein digestion, nitrogen metabolism	(Nayak 2010, Llewellyn et al. 2014b, Gajardo et al. 2016, Tarnecki et al. 2017, Rudi et al. 2018)
	*Acanthurus triostegus* (surgeonfish)	Wild	16S rRNA gene amplicon sequencing	Herbivorous (algae)	Firmicutes, Bacteroidetes, Cyanobacteria	*Epulopiscium, Acinetobacter, Arcobacter, Arthrospira, Brevinema, Cetobacterium, Fusobacterium, Methylobacterium, Photobacterium, Pelomonas, Vibrio, Pseudoalteromonas*	Algal polysaccharide breakdown, SCFA production	(Miyake et al. 2015, Tarnecki et al. 2017, Parata et al. 2020)

#### Freshwater fish gut microbiome

Based on the next-generation sequencing (NGS) technique, diverse groups of microbes have been detected in freshwater fishes. The core microbiomes are resistant to variation in diet and rearing density as claimed by researchers who experimented on GI microorganisms of *Onchorhynchus mykiss* (Wong et al. 2013). However, an alteration in diet can cause a change in the health status of fish (Wong et al. 2013). In herbivorous and omnivorous fish, the breakdown of cellulose can be enhanced by microbes, such as *Bacillus circulans* and *B. megaterium* (Saha et al. 2006). A study conducted on *Carassius auraus gibrlio* concluded that the first phylum of the microbe to develop in the gut is Proteobacteria (Li et al. 2017). However, the actual reason behind this fact is still unknown. We can assume that proteobacteria in environmental water enable the ability to interact with the host as bacteria are ubiquitous in water and are found to be the most abundant and diverse. They play an important role in nutrient cycling, decomposition, and organic matter breakdown. Common bacterial phyla found in water include Proteobacteria, Actinobacteria, Bacteroidetes, Cyanobacteria, and Firmicutes (Cottrell et al. 2005, Jordaan and Bezuidenhout 2013, Xia et al. 2013, Savio et al. 2015, Brar et al. 2023). Proteobacteria are the most dominant gut species in freshwater fish followed by Firmicutes, Actinobacteria, and Bacteroidetes (Wu et al. 2012). *Actinobacteria* spp. are well-known producers of secondary metabolites such as hydrolytic enzymes, e.g. amylase, protease, and lipase. Studies have revealed that *Actinobacteria* spp. play an important role in the fermentation of a large variety of oligosaccharides in the gut (Ventura et al. 2007). *Fusobacteria* spp. are most frequent in freshwater fishes (Kim et al. 2021). The freshwater fish gut is dominated by the species of *Enterobacter, Aeromonas*, and *Acinetobacter* (Cahill 1990, Mondal et al. 2008, Tsuchiya et al. 2008, Deb et al. 2020, Suescun-Sepulveda et al. 2023). Intestinal microflora also includes species of *Escherichia, Klebsiella, Proteus, Serrata, Aermonas, Alcaligenes, Eikenella, Bacillus, Listeria, Propionibacterium, Bacteroides, Citrobacter freundii, Hafnia alvei, Cytophaga/Flexibacter, Staphylococcus, Mycoplasma, Streptococcus, Lactococcus, Peptostreptococcus, Deefgea, Cetobacterium, Moraxella*, and *Pseudomonas* (Austin 2002, Brown et al. 2018, Hernández et al. 2021, Singh et al. 2021 ).

A comparison of the gut microbiome of rainbow trout (*Onchorhyncus mykiss*) and grass carp (*Ctenopharyngodon idella*) was done to better understand the variation in fish gut microbiome (Table 3). Based on their habitat, feeding habits, and access to freshwater, the species were chosen.

Rainbow trout is a freshwater carnivorous fish. It feeds on a wide variety of aquatic insects and crustaceans as well as small fish and even land insects that wash up on the surface of the water. Their diet can vary depending on where they live and what food sources are available (Huyben et al. 2018). Rainbow trout prefer chilled water having temperatures from 10 to 15°C. They may seek out certain parts of their habitat that have optimal temperature ranges. Several bacterial phyla, including Proteobacteria, Firmicutes, Bacteroidetes, and Actinobacteria, often dominate the gut microbiome of rainbow trout (Betiku et al. 2023). However, these phylas’ relative abundance can change. *Aeromonas, Pseudomonas, Acinetobacter, Shewanella, Clostridium*, and *Bacteroidetes* are the common genera discovered in rainbow trout gut. *Mycoplasma, Cetobacterium, Lactococcus, Lactobacillus, Leuconostoc, Ureaplasma*, and *Propionibacterium* were reported as an abundant genus (Llewellyn et al. 2014, Betiku et al. 2023). The gut microbiota in rainbow trout helps with digestion, modulating the immune system, and potentially protecting against pathogens. Some intestinal bacteria help in the digestion and utilization of complex polysaccharides and the synthesis of vitamins (Mondal et al. 2008, Li et al. 2016, Podell et al. 2023, Qi et al. 2023b).

Grass carp like freshwater habitats such as rivers, lakes, ponds, and reservoirs. Because their diet comprises primarily of plant material, they are usually found in places with abundant aquatic vegetation. Grass carp are noted for their herbivorous feeding habits, in which they consume a variety of aquatic plants (Ray et al. 2012). It can tolerate a wide range of temperatures but prefers warmer water. Temperatures ranging from 20 to 30°C are ideal. Different bacteria reported from grass carp gut microbiome include *Aeromonas, Bacillus, Clostridium, Bacteroides*, and lactobacilli. *Streptococcus, Lactobacillus, Flavobacterium, Veillonella, Pseudomonas, Anoxybacillus, Citrobacter, Clostridium*, and *Leuconostoc* were reported as abundant microbial genera in the *Ctenopharyngodon idella* intestine (Wu et al. 2012, Llewellyn et al. 2014). These occurrences and family abundance can be modified by factors such as food and environmental conditions.

The contrast underscores how diet and environmental factors affect the gut microbiomes of both species. The carnivorous tendencies and preference for cool water of rainbow trout have led to the development of a gut microbiome that is well adapted for digesting protein and fat efficiently, as well as for maintaining robust immune defences. On the other hand, the grass carp’s plant-based diet and preference for higher water temperatures help to create a digestive system full of beneficial bacteria that specialize in breaking down tough plant fibers and producing important nutrients from plants.

#### Marine water fish gut microbiome

The higher concentration of salt in water creates a challenging environment for fish; similarly, there is a possibility of variation in the environmental microbes. Although at the phylum level Firmicutes, Proteobacteria, and Actinobacteria are the most abundant species in the fish gut, at a lower taxonomic level, variations are observed. The marine fish intestinal flora consists of dominant species of *Vibrio, Pseudomonas, Achromobacter, Corynebacterium, Flavobacterium*, and *Micrococcus* (Cahill 1990, Izvekova et al. 2007, Ou et al. 2021) as well as *Aeromonas* spp., *Alcaligenes* sp., *Alteromonas* sp., *Micrococcus* sp., *Carnobacterium* sp., *Flavobacterium* sp., *Photobacerium* sp., *Pseudomonas* spp., *Staphylococcus* sp., and *Vibrio* sp. (Austin 2002, Izvekova et al. 2007, Huang et al. 2020, Ou et al. 2021), whereas in freshwater fish the composition varies as shown in Figs 3, 4, and 5.

A comparison of the gut microbiomes of Atlantic salmon (*Salmo salar*) and surgeonfish (*Acanthurus triostegus*) was done to better comprehend the variations in fish gut microbiota (Table 3). The species were chosen based on their habitat, feeding habits, and access to saltwater (Egerton et al. 2018, Huang et al. 2020, Ou et al. 2021, De Marco et al. 2023).

Atlantic salmon spend most of their lives in the Atlantic Ocean. They are recognized for their anadromous habit, which means that they travel from freshwater to the ocean and return at various phases of their lives. Atlantic salmon prefer cold, well-oxygenated waters. Their adaptation to varied environmental circumstances is demonstrated by their capacity to live in both freshwater and saltwater (Morales et al. 2022). Atlantic salmon are opportunistic eaters in the ocean, devouring a wide range of marine creatures. *Pseudomonas, Janthinobacterium, Stenotrophomonas, Delfia, Herbaspirillum, Burkholderia, Sphingomonas, Propionibacterium, Ochrobacterium, Variovorax, Microbacterium, Phyllobacterium, Rhodococcus*, and *Acinetobacter* are the abundant genera in the *Salmo salar* GI tract (Llewellyn et al. 2014, Gajardo et al. 2016).

*Acanthurus triostegus*, sometimes known as the Convict Tang, is commonly found in tropical marine settings with warm water temperatures. It thrives at the temperatures found in coral reef ecosystems. They are herbivorous and mostly eat algae. *Epulopiscium, Acinetobacter, Arcobacter, Arthrospira, Brevinema, Cetobacterium, Fusobacterium, Methylobacterium, Photobacterium, Pelomonas, Vibrio*, and *Pseudoalteromonas* are the most prevalent microbial genera found in the digestive tract of surgeonfish (Miyake et al. 2016, Ngugi et al. 2017a, Parata et al. 2020).

The gut microbiomes of both Atlantic salmon and Convict Tang species are varied and can be affected by what they eat and the surroundings they live in. Atlantic salmon, as anadromous fish, do well in cold waters and eat a range of marine animals because they are opportunistic feeders. The bacteria found in their gut microbiome, such as *Pseudomonas* and *Burkholderia*, help with absorbing nutrients (Moore et al. 2006, Wang et al. 2018). On the other hand, Convict Tang species live in tropical marine habitats and mainly eat algae. The bacteria found in their gut microbiome, such as *Epulopiscium* and *Vibrio*, are specifically designed to break down algal material (Thompson and Polz 2006, Miyake et al. 2016, Ngugi et al. 2017b, Sampaio et al. 2022). This points out how diet and environmental conditions affect the composition of the gut microbiome, with Atlantic salmon containing bacteria that digest protein and Convict Tang having bacteria that degrade algae, which helps them thrive in their specific diets and habitats.

### Impact of environment on fish gut microbiome

The fish gut microbiome is critical to their health, development, and overall well-being. Quality of water, habitat and diet can all have a substantial impact on—composition and function of their gut microbiome (Sullam et al. 2012, Wong and Rawls 2012, Dehler et al. 2017, Huyben et al. 2018, Huang et al. 2020, Kim et al. 2021, Leeper et al. 2021, Karlsen et al. 2022, Brar et al. 2023, Herrera et al. 2023, Yin et al. 2023a, Kanika et al. 2024). Recent studies on the gut microbiota of tilapia concluded that the optimal composition and functions of the gut microbiota are not always accurately represented by the highest growth outcomes of the host (Ou et al. 2024). The negligent inclusion of macronutrients negatively affects the gut microbiota. Hence, it is important to take into account both growth performance and gut microbiota when assessing specific macronutrients (Ou et al. 2024). A study conducted on rainbow trout by changing the water temperature and diet found a decrease in the number of important microbes (order Lactobacillales) in the gut (Huyben et al. 2018). The studies also assumed that a high proportion of gut bacteria represented by *Mycoplasma* sp. (phylum Tenericutes) is nutrient-dependent, which means that these bacteria develop only in the presence of specific nutrients, because many studies on the same species did not report this bacteria (Huyben et al. 2018). A study was conducted in which the intestinal microbiota of Atlantic salmon was evaluated in two different habitats, namely a recirculated aquarium facility and an open freshwater loch cage. The researchers found variations in the composition of the microbiome such as the greater presence of phylum Tenericutes in aquarium fish samples, whereas Proteobacteria were more abundant in loch samples; similarly, Mycoplasmataceae (phylum Tenericutes) was the second most common family in aquarium fish samples but less common in loch fish samples (Dehler et al. 2017). A study conducted on yellowtail kingfish found that an increase in water temperature (26°C) caused changes in the microbial communities of young yellowtail kingfish, influencing their growth trajectory and immunological condition (Horlick et al. 2020). Temperature is essential in determining the composition of the gut microbiome in humans and fish (Wang et al. 2018, Sepulveda and Moeller 2020, Larios-Soriano et al. 2021). Elevated temperatures may boost metabolic rates, aiding heat-resistant microbes and harmful bacteria, whereas lower temperatures can slow microbial metabolism and benefit cold-adapted species (Abram et al. 2017, Huyben et al. 2018, Ghosh et al. 2022). Fish are significantly affected by temperature changes because they are cold-blooded, which can impact their health and size (Wu et al. 2022). Temperature, diet, and habitat all affect the gut microbiome of fish, leading to changes in metabolism, immune response, and overall health (Collazos et al. 1994, Horlick et al. 2020, Sepulveda and Moeller 2020, Li et al. 2023). Keeping the ideal water temperature is crucial in aquaculture to improve gut bacteria health, boost fish growth, and strengthen disease defences.

**Figure 6.**
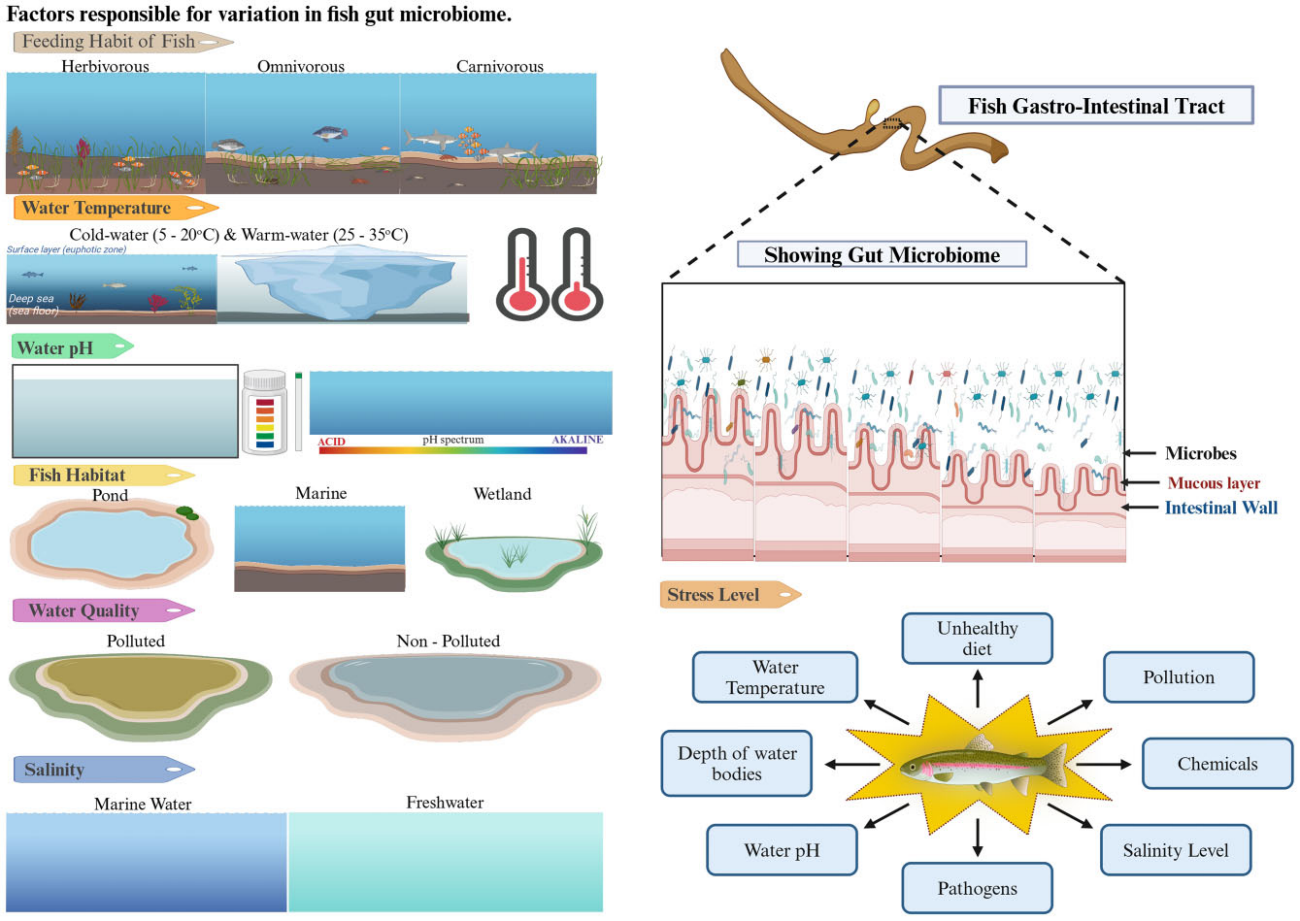
Some factors responsible for variation in fish gut microbiome.

Our comparative study also indicates that environmental factors cause changes in gut microbial composition in the host. In Table 3, the most abundant bacterial genera are found to be different due to their different habits, habitats, and species they belong to. It is assumed that the gut microbiome helps in the adaptation of the host to different environments and requirements. Multiple factors such as environment, diet, host immunity, microbes, habit, habitat, water quality, etc. can make a host capable of sustainable survival. A clear visualization is presented in Fig. 6, which shows some factors responsible for variation in fish gut microbiome (Al-Harbi and Uddin 2004, Escalas et al. 2021, Kim et al. 2021, Podell et al. 2023, Bharti et al. 2023, Herrera et al. 2023, Sadeghi et al. 2023, Small et al. 2023, Viver et al. 2023).

## Conclusion and future directions

There are notable shifts in the microbial communities of the gut microbiomes of marine and freshwater fish. These variations are influenced by both the diet and the surrounding water sources. In spite of these differences, there are certain resemblances in the gut microbiomes of marine and freshwater fish. *Aeromonas, Vibrio, Pseudomonas*, and other species are present in the GI tract of marine and freshwater fish, contributing to nutrition metabolism, fermentation, and overall gut health. Different microbes present in the intestines of fish create bioactive compounds by using specific genes. Examples include *cobA, cobG*, and *cobT* for Vitamin B_12_ creation; *but* and *buk* for short-chain fatty acid output; *iucA* and *pvd* for obtaining iron; *lantA* and *nisA* for generating antimicrobial peptides; *PKS* and *NRPS* routes for producing antibiotics and pigments; *luxI* and *luxR* for regulating population density; and *tnaA* for creating indole. These genetic mechanisms are essential for preserving the health of the fish gut and protecting against infections. The gut microbiome of fish is made up of symbiotic connections between the host and microbes, with bacteria breaking down nutrients and generating Vitamin B_12_. The immune system accepts beneficial bacteria and suppresses harmful ones, as quorum sensing enables bacteria to communicate. Microbes vie for space and nutrients, leading to the development of specific ecological niches. Antagonistic interactions in a balanced microbiome consist of creating antimicrobial substances, resource competition, and interrupting pathogen communication. Comprehending the fish microbiome is essential for grasping the intricate connections between microbes and their hosts. Continuing research is providing an understanding of the functional roles of these microorganisms and their effects on the health of fish in different aquatic environments. As advancements are made in the field, new findings can influence aquaculture, the management of fisheries, and our comprehension of aquatic ecology. More research is required to comprehend how host–microbiome interactions coevolve and adapt, as well as the specific roles of certain microorganisms in processing nutrients and regulating the immune system. It is important to study the gut microbiomes of wild fish populations in order to understand their natural microbial communities and ecological functions besides focusing on aquaculture or laboratory fish. This comparative study will help increase the understanding of aquatic microbiology and develop techniques to enhance the health and sustainability of fish populations in various aquatic habitats.

## Supplementary Material

fiae169_Supplemental_File
