# Supplementary material for: A review on comparative analysis of marine and freshwater fish gut microbiomes: insights into environmental impact on gut microbiota
Source: FEMS Microbiol Ecol. 2024 Dec 24;101(1):fiae169. doi: 10.1093/femsec/fiae169 (PMC11730441; doi:10.1093/femsec/fiae169)
Supplement: fiae169_Supplemental_File [file fiae169_supplemental_file.docx]

**Supplementary Table 1.** Species diversity of fishes studied and the number of bacterial taxa found in their intestines

| **Sl. No.** | **Species** | **Salinity preference** | **Number of bacterial taxa** |
| --- | --- | --- | --- |
|  | *Abramis brama* | F | 3 |
|  | *Anguilla japonica* | F | 10 |
|  | *Callionymus sp.* | M | 2 |
|  | *Carassius auratus* | F | 23 |
|  | *Carassius Carassius* | F | 10 |
|  | *Clupea harengus* | M | 6 |
|  | *Conger japonicus* | M | 2 |
|  | *Ctenopharyngodon idella* | F | 8 |
|  | *Cyprinus carpio* | F | 23 |
|  | *Dicentrarchus labrax* | M | 6 |
|  | *Ditrema temmincki* | M | 2 |
|  | *Esox lucius* | F | 4 |
|  | *Gadus morhua* | M | 5 |
|  | *Girella punctata* | M | 2 |
|  | *Gobio gobio* | F | 6 |
|  | *Gymnocephalus cernus* | F | 4 |
|  | *Hermosilla azurea* | M | 4 |
|  | *Hypophthalmichthys molitrix* | F | 2 |
|  | *Ictalurus panctatus* | F | 17 |
|  | *Labeo rohita* | F | 3 |
|  | *Mallotus villosus* | M | 1 |
|  | *Melanogrammus aeglefinus* | M | 6 |
|  | *Microstomus kitt* | M | 6 |
|  | *Morone saxatilis* | M | 4 |
|  | *Mugil sp.* | M | 2 |
|  | *Oncorhynchus mykiss* | F* | 26 |
|  | *Oncorhynchus spp.* | F* | 5 |
|  | *Oreochromis niloticus niloticus* | F | 12 |
|  | *Oreochromis niloticus niloticus* Ⅹ *Oreochromis aureus* | F | 18 |
|  | *Ostracion cubicus* | M | 2 |
|  | *Perca fluviatilis* | F | 6 |
|  | *Phoxinus sp.* | F | 3 |
|  | *Plecoglossus altivelis* | F* | 9 |
|  | *Pollachius virens* | M | 1 |
|  | *Pomatomus saltatrix* | M | 7 |
|  | *Pseudolabrus japonicus* | M | 2 |
|  | *Raja spp.* | M | 10 |
|  | *Rutilus rutilus* | F | 9 |
|  | *Salmo salar* | F* | 2 |
|  | *Salmo trutta* | F* | 9 |
|  | *Salvelinus alpinus* | F* | 22 |
|  | *Sardinalla spp.* | F* | 7 |
|  | *Scomber spp.* | M | 2 |
|  | *Seriola quinqueradiata* | M | 3 |
|  | *Sillago japonicus* | M | 2 |
|  | *Solea solea* | M | 9 |
|  | *Sparus aurata* | M | 6 |
|  | *Stizostedion lucioperca* | F | 3 |
|  | *Takifugu nophobles* | M | 3 |
|  | *Tinca tinca* | F | 1 |
|  | *Trachurus spp.* | M | 7 |

Note: (F) Freshwater, (M) Marine
*The species have anadromous and semi-anadromous forms.

**Source:** (Izvekova, Izvekov and Plotnikov 2007)
